# Direct Inference of SNP Heterozygosity Rates and Resolution of LOH Detection

**DOI:** 10.1371/journal.pcbi.0030244

**Published:** 2007-11-30

**Authors:** Xiaohong Li, Steven G Self, Patricia C Galipeau, Thomas G Paulson, Brian J Reid

**Affiliations:** 1 Division of Public Health Sciences, Fred Hutchinson Cancer Research Center, Seattle, Washington, United States of America; 2 Division of Human Biology, Fred Hutchinson Cancer Research Center, Seattle, Washington, United States of America; 3 Department of Medicine, University of Washington, Seattle, Washington, United States of America; 4 Department of Genome Sciences, University of Washington, Seattle, Washington, United States of America; Lilly Singapore Centre for Drug Discovery, Singapore

## Abstract

Single nucleotide polymorphisms (SNPs) have been increasingly utilized to investigate somatic genetic abnormalities in premalignancy and cancer. LOH is a common alteration observed during cancer development, and SNP assays have been used to identify LOH at specific chromosomal regions. The design of such studies requires consideration of the resolution for detecting LOH throughout the genome and identification of the number and location of SNPs required to detect genetic alterations in specific genomic regions. Our study evaluated SNP distribution patterns and used probability models, Monte Carlo simulation, and real human subject genotype data to investigate the relationships between the number of SNPs, SNP HET rates, and the sensitivity (resolution) for detecting LOH. We report that variances of SNP heterozygosity rate in dbSNP are high for a large proportion of SNPs. Two statistical methods proposed for directly inferring SNP heterozygosity rates require much smaller sample sizes (intermediate sizes) and are feasible for practical use in SNP selection or verification. Using HapMap data, we showed that a region of LOH greater than 200 kb can be reliably detected, with losses smaller than 50 kb having a substantially lower detection probability when using all SNPs currently in the HapMap database. Higher densities of SNPs may exist in certain local chromosomal regions that provide some opportunities for reliably detecting LOH of segment sizes smaller than 50 kb. These results suggest that the interpretation of the results from genome-wide scans for LOH using commercial arrays need to consider the relationships among inter-SNP distance, detection probability, and sample size for a specific study. New experimental designs for LOH studies would also benefit from considering the power of detection and sample sizes required to accomplish the proposed aims.

## Author Summary

More than 99% of each person's genome is identical to everyone else's. Many of the differences involve single base pairs, termed single nucleotide polymorphisms (SNPs). SNPs are used as genetic markers to facilitate identification of disease-causing genes, as well as in cancer studies by aiding in determining which regions of the genome may be lost (LOH) or amplified during neoplastic progression. One drawback to SNPs is their low informativity: a SNP is only informative if it is polymorphic on the two different alleles found on each chromosome of a pair; and if there is not an informative SNP in the region of genome of interest, it is impossible to detect alterations occurring there through LOH. A common solution to this problem is to use arrays containing hundreds of thousands of SNPs to ensure adequate coverage, but for many studies this is prohibitive on a cost and sample amount basis. In addition, SNP distribution itself can constrain the size of loss that can be reliably detected at the population level. We examined the relationship between chromosome loss sizes and detection probability of LOH genome-wide. The study provides useful information for researchers designing LOH-related studies and evaluating results obtained from such studies.

## Introduction

Single nucleotide polymorphisms (SNPs) are common DNA sequence variations and have been widely investigated for their roles in disease causation [1] or association [2,3], heterogeneous responses to drug therapies [4–6], genetic linkage analysis [7,8], and evolutionary biology [9,10]. This has led to the characterization of whole-genome patterns of a large number of common SNPs in a few ethnic groups [11]. Distinct from constitutive genome studies, SNPs have also been used extensively to study the somatic development of cancer [12] (also see review by Engle, et al. [13]). Alterations of the copy number of DNA sequences (DNA amplification or deletion) and those that result in a loss of genetic information (loss of heterozygosity; LOH) occur frequently in neoplastic tissues and tumors, and changes in the copy number or heterozygosity of SNPs allow these alterations to be detected and mapped in the genome. Low density, whole genome analyses have previously been sufficient to allow gross characterization of critical genetic alterations that occur during neoplastic progression. However, much finer-scale mapping of these alterations is frequently required both for furthering basic understanding of the genetic events that occur during progression to cancer and for developing diagnostic tests with sufficient sensitivity and specificity for translation into clinical practice. In addition, commercial high-density SNP platforms tend to be both expensive and biospecimen-intensive, making them impractical for high-throughput, fine-scale mapping of specific chromosomal regions. The alternative is to develop a custom panel of SNPs that can characterize the genomic region of interest.

Detection of LOH requires SNPs to be heterozygous (i.e., informative). In the largest public SNP database, dbSNP (http://www.ncbi.nlm.nih.gov/SNP), the heterozygosity (HET) rates estimated for a substantial number of SNPs have large estimated variances, likely due to small sample sizes, among other reasons [14]. Using SNPs with large HET rates variances may lead to ambiguous experimental results (e.g., an under-powered study). A better understanding of how the distribution of SNPs in the genome and the variance of SNP HET rates affect the ability of a panel of SNPs to detect LOH would allow improved design of SNP-based assays for somatic genetic alteration studies. Statistical models for classifying subjects by LOH profile that take into account noninformative markers have been developed [15]. We used real genotype data to investigate the relationship between detection probabilities (resolution) and LOH sizes using all currently characterized SNPs in Hapmap, a relationship that is closely related to sample size and power calculations in LOH detection experimental design. As well, the study evaluates the key factors governing the selection of a group of SNPs for designing custom assays for particular chromosomal regions. Specifically, we first evaluated the variances in SNP HET rate estimation currently reported in the dbSNP database, and then addressed sample size issues related to directly inferring SNP HET rates for the purpose of selecting SNPs for LOH detection. We propose two statistical approaches to determine the minimum number of individuals in a population that would need to be examined to determine if a SNP HET rate was above or below a specified threshold. Finally, we evaluate the relationships between the number of SNPs, SNP HET rates, and sensitivity (resolution) for detecting LOH using real whole-genome genotype data.

## Results

**Figure 1:**
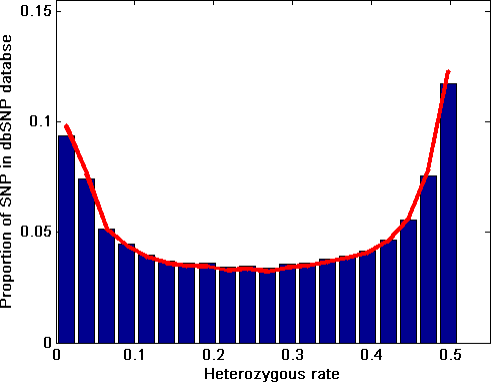
Frequency Distribution Pattern of Estimated Average SNP HET Rates in the dbSNP Database Blue bars are the distribution of SNP HET rates in dbSNP; red line is fitted line. Chi-square goodness of fit test (with 20 bins) for fitting a beta distribution was not rejected at *α* = 0.01 level.

**Figure 2:**
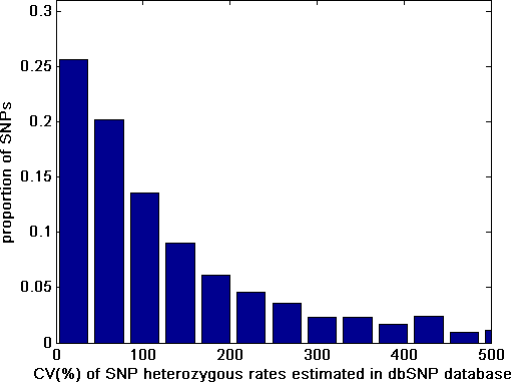
Frequency Distribution of Estimated CVs of SNP HET Rates in the dbSNP Database The *x*-axis is truncated at CV > 500% for illustrative reasons even though SNPs with higher CVs were included in the actual distribution analysis. About 30% of SNPs have an estimated CV of ≤50%, less than 13% of SNPs had an estimated CV of ≤20%, and less than 4% of the SNPs had a low CV (≤5%).

The frequency distribution of the average SNP HET rates for each SNP reported in the dbSNP database is shown in Figure 1. The genome-wide mean HET rate is 0.263 (SD = 0.171). The observed pattern was similar to a beta distribution, although the data do not exactly fit a formal beta distribution. Figure 2 shows the distribution of the estimated coefficient of variation (CV = SD/Mean%) of SNP HET rates in the dbSNP database.

These results indicate that a significant number of the SNPs in dbSNP have large estimated variances, which would not provide enough precise information for designing studies requiring the accurate estimation of SNP HET rates (i.e., those using SNPs for LOH detection for molecular diagnoses). Traditionally, for diallelic alleles with *p*_1_ and *p*_2_ allele frequencies, the HET rate could be estimated as *h_r_* = 2*p*_1_ *p*_2_, although this formula is appropriate only for alleles in HWE. Another approach, which is robust to HWE assumptions, is to estimate the HET rate (and its variance) directly by population allele frequencies [16]. This method requires large sample sizes in order to achieve accurate estimation of HET rates. Here we consider the case where the HET rate is measured directly using techniques like DNA sequencing, microarray analysis, Pyrosequencing, or MALDI-TOF.

Using hypothetical parameters for true HET rates and sample sizes, we first show the relationships among true HET rates, estimated HET rates, their estimated variances, and sample sizes using the score method with continuity correction (exact binomial method may result in larger CI) (Table 1). The variance of HET rates could be estimated by (*h_r_*(1 − *h_r_*))/(*N* − 1), where *N* is the sample size. Table 1 shows that even with moderately large sample size (e.g., *N* = 100), the confidence interval or CVs are quite large for all values of HET rates listed, particularly for lower HET rates (some upper bounds of the CI even exceeded the theoretical maximum value of *h_r_* = 0.5). With 500 subjects tested, the estimated CI and variance are small, but such a sample size is prohibitively large for many studies.

**Table 1:**
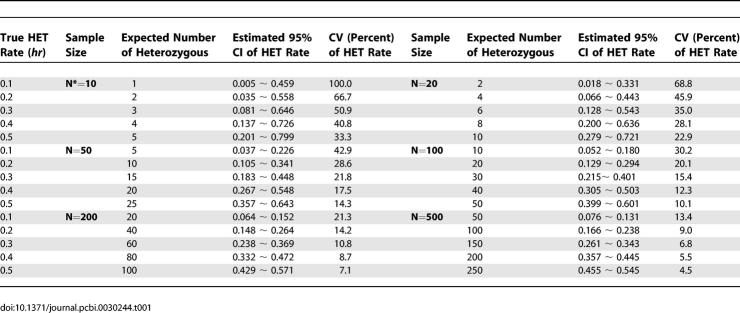
Relationship between Sample Size and Estimation of SNP Heterozygous Rates

We introduce two different approaches to deal with the unrealistically large sample size requirement. In using SNPs to evaluate LOH in a specific chromosomal region, it is desirable that the HET rates of selected SNPs used in the region be higher than a specific value to increase the probability that at least one SNP will be informative for each patient. Therefore, the question is to test the statistical hypothesis for the HET rate of a specific SNP *h_rs_* versus a prespecified HET rate value *h_r_*_0_ (i.e., *H*_0_: *h_r_* ≥ *h_r_*_0_ versus *H_1_*: *h_r_* < *h_r_*_0_). With a given power and sample size *n*, we have:

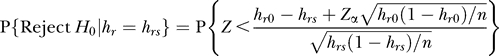



To get sample size, we have: 
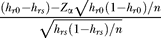
 = *Z_β_*, where *Z_α_* and *Z_β_* are the 100(1−*α*)th and 100(1−*β*)th percentile of the standard normal distribution.

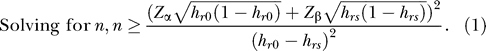



Using Equation 1, Table 2 shows the sample sizes needed for testing whether the HET rate of a given SNP is significantly higher than a desired threshold. The sample size required to reject *H*_0_ is reasonably small in most cases; e.g., when *h_r_*_0_ = 0.2, and *h_rs_* = 0.35, only 50–72 subjects need to be tested. However, when a SNP HET rate *h_rs_* is near the desired threshold value *h_r_*_0_, the required sample size becomes much larger.

**Table 2:**
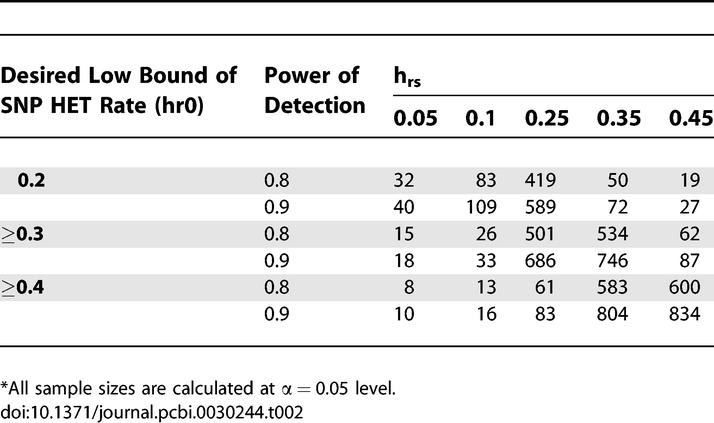
Sample Sizes* for Testing SNP Heterozygous Rate at Different Thresholds

Table 2 utilizes a fixed sample size method that is easy to use, but may not be optimal when considering the number of subjects needed. A sequential sampling technique based on the sequential probability ratio test (SPRT) [17] can be used for directly inferring SNP heterozygous rates. With this method, samples are tested one by one and a decision will be made to determine whether or not the HET rate of a SNP has reached a prespecified value after each sample is tested. This method generally requires less time and reagents and conserves biospecimens that are frequently unique and difficult to obtain. Specifically, SPRT tests the SNP HET rate *h* using the hypothesis *H_0_: h = h*_0_ versus *H*_1_ *: h = h_1_*, (*h*_1_ *< h*_0_). The likelihood ratio is

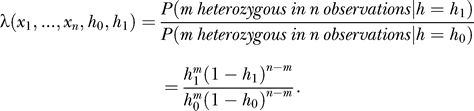



For type I error (false positive) level *α*, and type II error (false negative) level *β*, (power = 1 − *β* ), it has been shown that sample testing should continue if *ln* 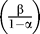 < *ln*(*λ*(*x_1_, …, x_n_, h*_0_ *, h*_1_)) < *ln* 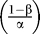 ; if *ln*(*λ*) reaches or passes beyond the two bounds, then sample testing should stop. The hypothesis *H*_0_ will be accepted when *ln* 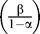 ≤ *ln*(*λ*), or *H*_0_ will be rejected and *H*_1_ accepted when *ln*(*λ*) ≥ *ln* 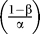 . In this process, the total number of samples tested is a random variable based on the distribution specified by parameters *h*_0*,*_ *h*_1_, *α*, *β*, and the underlying HET rate *h* of a specific SNP. In the SPRT approach, for fixed *h*, *α*, and *β*, the ASN (average sample number) depends on *h*_0_ and *h*_1_. Table 3 shows simulation results for testing *h*_0_ = 0.3, and *h*_1_ = 0.2 against various true (sample) SNP HET rates *h*. For example, if the true SNP HET rates under testing are *h* = 0.4 or above, approximately 15 to 40 subjects need to be tested, on average, to make a decision on whether *h = h*_0_, and, under the most optimistic situations, only four subjects are necessary to determine the HET rate regarding hypothesis *H*_0_. Depending on the goals of a study, the SPRT method could be used to significantly reduce the testing sample size required for SNP HET rate inference (e.g., compare to values in Table 1).

**Table 3:**
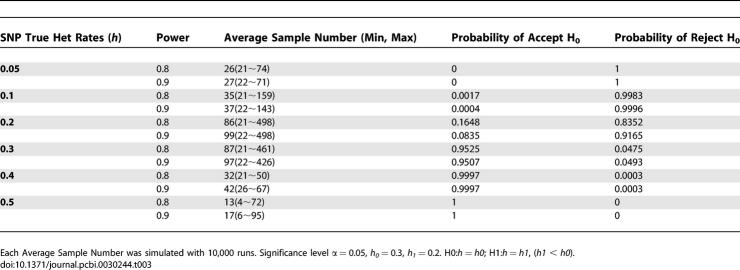
Average Sample Number of Sequential Probability Ratio Test Method for SNP HET Rate Test

**Figure 3:**
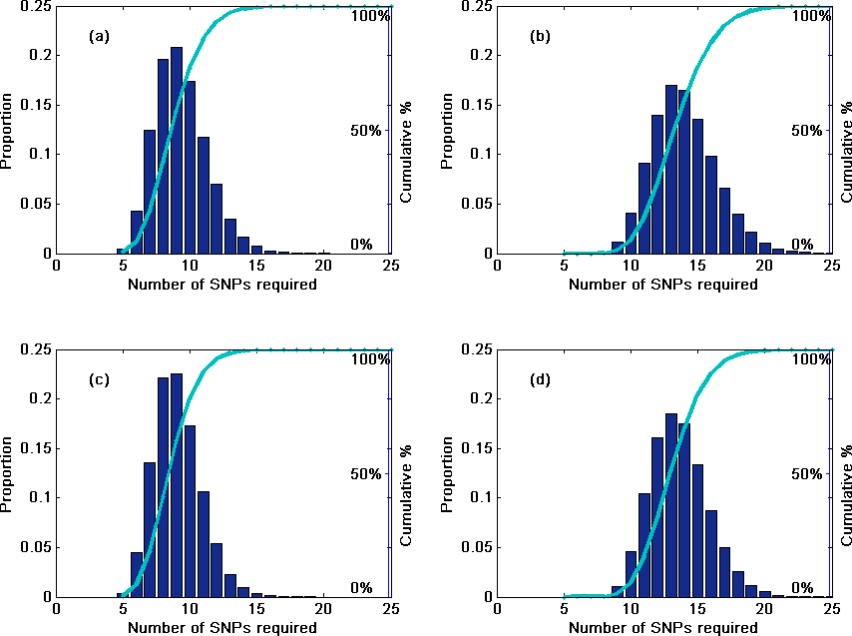
Probability Density Function (Bars) and Cumulative Density (Lines) of the Number of SNPs Needed To Have at Least One Heterozygous SNP Based on Simulation Results Using the Distribution Shown in Figure 1 At *α* = 0.01 level, (A) and (C) are for left-hand side of Equation 2 ≥ 0.95; (B) and (D) for ≥ 0.99, respectively. (A,B) Are the results of excluding SNPs with HET rates > 0.5. (C,D) Are the results without the exclusion. The required number of SNPs *k_i_* can be estimated based on the cumulative density distribution function (cdf): [1 − P(*k* ≤ *k_i_*) ] ≤ α. The simulation shows that if SNPs were randomly used for LOH detection, then *k_i_* = 15 for threshold = 0.95; and *k_i_* = 20 for threshold = 0.99 (both were calculated at *α* = 0.01 level for cdf).

We also examined the number of SNPs needed for reliable detection of LOH for random chromosomal regions of a specific length assuming the SNP HET rate distribution shown in Figure 1. If SNPs are being used to effectively detect the loss of a chromosomal segment, the segment should contain at least one or more heterozygous SNPs. If all SNPs have an identical HET rate *h_t_* (0 < *h_t_* ≤ 0.5), then *k* SNPs are needed such that 1 − (1 − *h_t_*)^k^ ≥ threshold (i.e., threshold = 0.95 or 0.99) to guarantee at least one or more heterozygous SNP will be in the lost segment. However, *h_t_* is not constant across all SNPs (Figure 1). Therefore, *k* SNPs are needed to have:

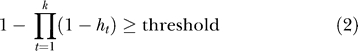
where the threshold (i.e., threshold = 0.95 or 0.99) is the probability of having at least one or more heterozygous SNP in the chromosome segment. Based on the distribution pattern of HET SNP rates (Figure 1), Monte Carlo simulation was used to estimate the number of SNPs needed (*k*) to satisfy Equation 2 at the *α* level (i.e., 0.05 or 0.01) which guarantees that the left-hand-side of Equation 2 will lie beyond the threshold (1 − *α*) 100% of the time. The probability density distribution of *k* is shown in Figure 3 based on the results of these simulations. Similarly, the simulation indicates that if SNPs with HET rates ≥0.3 are randomly selected for use, then the required number of SNPs (*k*) is 10, and for a SNP HET rate ≥0.4, the required number of SNPs (*k*) is 9 (both calculated at *α* = 0.01 level using the cumulative density function and threshold = 0.95, unpublished data).

**Figure 4:**
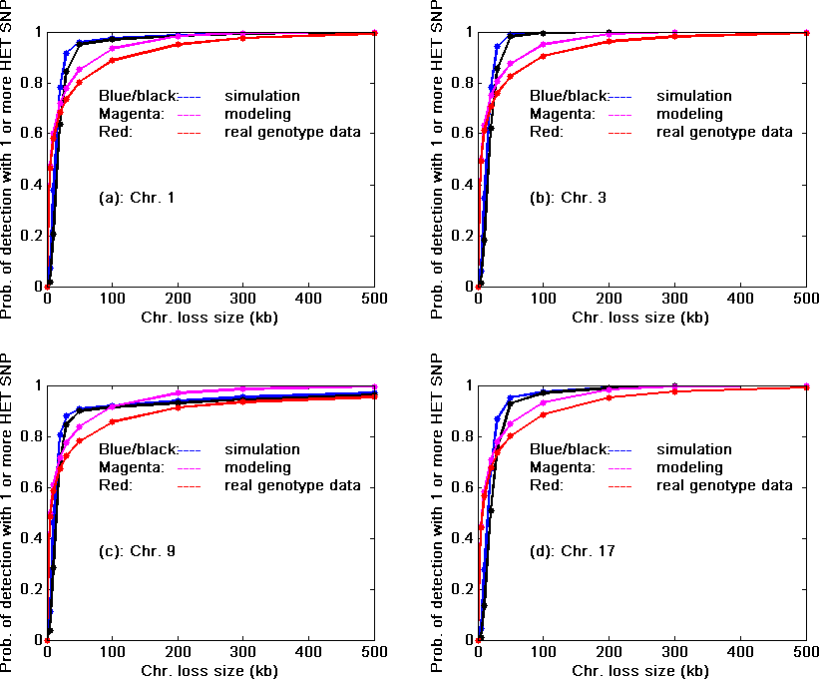
Relationship between Size of Chromosome Loss (kb) and Probability of Detection of LOH Assuming Use of All Chromosome 1, 3, 9, and 17 SNPs in HapMap Blue and black lines are the simulated results using HET rate distribution pattern in dbSNP (Figure 1) with the assumptions of successful detection if *k* = 15 (95%) or 20 (99%) SNPs per lost segment as shown in Equation 2. Red lines represent the probability of detection of LOH using HET SNPs based on real genotype data of 90 patients in the CEU group of the HapMap. Magenta lines represent the probability of LOH detection based on fitted model (HET SNP distribution was fitted with negative binomial distribution) prediction. The simulation results indicated a detection probability of about 75%–85% for 30 kb loss size (blue, black); the probability of detection reaches 95% or higher when loss size is approximately 50 ∼ 60 kb or larger. The LOH size approximately has to be 250 kb or larger in order to achieve a 99% or higher detection probability (except for Chromosome 9 with slightly lower probability values). The results based on real genotyping data (red line) indicate a detection probability of about 70% for a 30 kb loss size; the probability of detection reaches 95% or higher when loss size is approximately 200 kb or larger, and the loss size has to be 450 kb or larger in order to achieve a 99% detection probability. The results based on model fitting (magenta lines) appear to be a good approximation of the results based on genotyping data (red lines).

Given the non-random distribution pattern of SNP HET rates in the genome, the next obvious question is how long (in base pairs) must a random chromosomal segment be to contain one or more heterozygous SNPs so that LOH is detected with a high probability (e.g., 0.95 or 0.99). Based on HapMap data, we used three approaches to ascertain this relationship, including simulation using the fitted dbSNP HET rate distribution pattern in Figure 1, modeling of the SNP HET rate distribution within various chromosome deletion sizes using a negative binomial distribution (model not shown), and random sampling along a chromosome based on real genotyping data. The results from the three approaches are shown in Figure 4 using Chromosomes 1, 3, 9, and 17, which frequently undergo alterations in many cancers, as examples.

**Figure 5:**
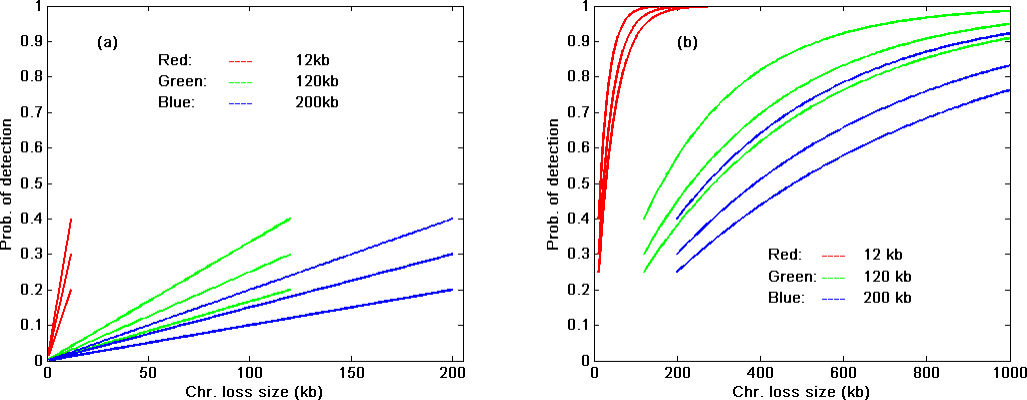
Relationship among Inter-SNP Distance, Size of LOH, and Probability of Detection of LOH with Heterozygous SNPs, Assuming an Even Distribution of SNPs (Red lines: inter-SNP distance = 12kb; green lines: inter-SNP distance = 120kb; blue lines: inter-SNP distance = 200 kb). For each color, the three lines from bottom to top correspond to SNP HET rates of 0.2 (bottom), 0.3 (middle), and 0.4 (top). (A) Shows the results when the chromosomal region being lost is smaller than the inter-SNP distance. For example, with a 100 kb region being lost and a 200 kb inter-SNP distance, the LOH detection probabilities are 8%, 15%, and 20% for 0.2, 0.3, and 0.4 SNP HET rates, respectively, (blue lines). The maximum detection probability is about 40% or less, depending on SNP HET rate. (B) Shows the results when the region of loss size is larger than the inter-SNP distance. For a 300 kb region of loss size and a 120 kb inter-SNP distance, the detection probability is about 40%, 60%, and 70% for SNP HET rates of 0.2, 0.3, and 0.4, respectively, in the calculation (green lines). As the region of loss increases, approaching 900 kb, the LOH detection probability will approach 0.9 or higher when the SNPs have a HET rate of 0.3 or higher. Similarly, with a 200 kb inter-SNP distance and a region of loss of 300 kb, the probabilities of detection of LOH are about 28%, 40%, and 52% for SNP HET rates of 0.2, 0.3, and 0.4, respectively (blue lines). If the inter-SNP distance is 12 kb, the detection probability of LOH is fairly high (more than 85%) when loss size is about 100 kb or longer (red lines). The results were based on the assumption that the SNPs selected and arrayed on the chips are evenly distributed on the chromosome, which gives the most optimistic detection probability for genome-wide screening. If the selected SNPs on a chip are not evenly distributed, the detection probability will be reduced. If all the current available SNPs are used (arrayed on a chip), the detection probabilities become the pattern as shown in Figure 4.

Many publications [11,18–20] have reported the mean/median distance between SNPs (inter-SNP distance) on specific arrays used in various studies. Therefore, we explored the relationships between inter-SNP distances, SNP HET rate, and detection probability of LOH to determine the chromosome segment size in base pairs required to have a reasonable chance of containing an informative SNP. Let *s* be the size (in nucleotide base pairs) of the DNA being lost on a chromosome, *d* the distance (in nucleotide base pairs) between two SNPs (inter-SNP distance), and *h_het_* the SNP HET rate, assuming the SNPs to be evenly distributed. If *s ≤ d*, the probability of the lost DNA segment containing a SNP can be estimated as *p* = 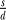 , and the probability of detecting of LOH with HET SNPs is *p_d_ = ph_het_* (Figure 5A). When *s* > *d*, the number of SNPs within the lost region is *k* = 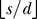 (_⌊ ⌋_ representing the largest integer equal to or smaller than *s/d*). The probability that at least one SNP is heterozygous can be estimated as *p_d_* = 1 − (1 − *h_het_*)^k^. The relationships are shown in Figure 5.

**Figure 6:**
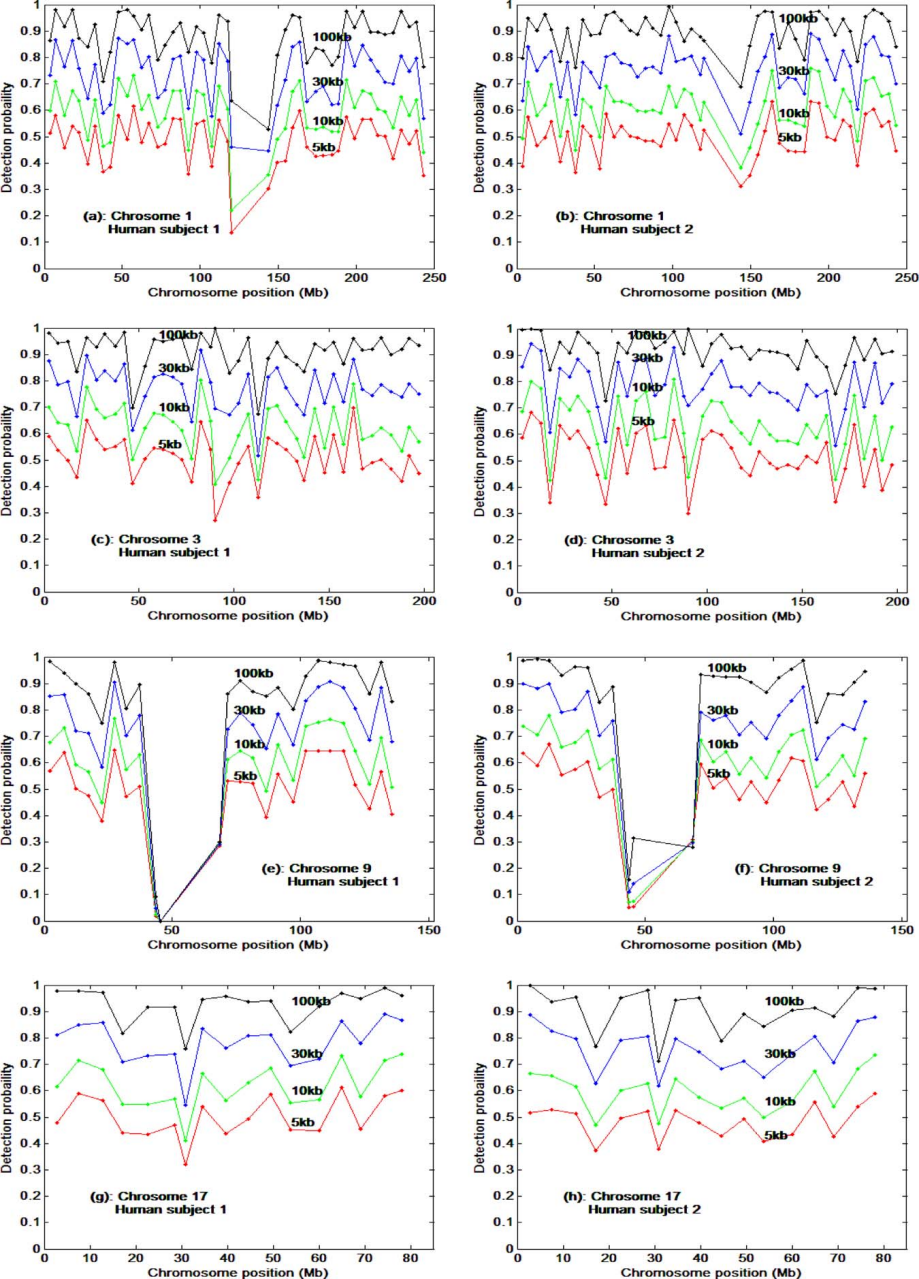
Spatial Distribution Pattern of LOH Detection Probabilities with Heterozygous SNPs on Chromosomes 1, 3, 9, and 17 for Various Loss Sizes The HapMap genotype data are from two randomly selected individuals from the CEU group.

Finally, we used a bootstrap method to randomly sample the heterozygous SNPs on Chromosomes 1, 3, 9, and 17 genotype data within a 500 kb window in two human subjects from the HapMap database. Figure 6 shows the spatial distribution of LOH detection probabilities with heterozygous SNPs on Chromosomes 1, 3, 9 and 17 for various loss sizes (5 kb, 10 kb, 30 kb, and 100 kb), assuming all known SNPs in that region were used. The mean of the detection probabilities of each loss size are very similar to the results shown in Figure 4.

## Discussion

Using SNPs for LOH detection is of great value for chromosomal instability studies and cancer risk prediction, but a better understanding of the resolution of the technique and how to select an informative panel of SNPs for a given application is needed. The variances of SNP HET rates are large for a large number of SNPs. In most cases, this is likely to be due to the small sample sizes used for estimation of allele frequencies in most cases. Differences in ethnic groups might also contribute to the variance of averaged HET rates. Relatively large sample sizes are needed to accurately estimate SNP HET rates using traditional methods. In order to reduce sample size for practical use, we presented two statistical methods that could be used to determine the number of individuals in the population that would need to be examined to determine if a SNP HET rate was above or below a specified threshold. The Monte Carlo simulation was performed on SNPs in dbSNP with HET rate estimation values ≤0.5 as well as all SNPs, with essentially no change in the conclusion of the study (Figure 3). Only 0.2% of the SNPs in dbSNP have a HET rate estimation higher than 0.5, some of which may be truly higher due to violations of Hardy-Weinberg equilibrium and some due to other factors such as estimation from a small sample size.

Based on specific study goals or technologies, more study specific methods such as truncated SPRT schemes [17] could potentially be used to minimize the sample size when the HET rate is close to the testing rate. In addition, since different human populations (e.g., Asian versus African versus European) may have different SNP distribution patterns [21–23], the sample size calculation methods may only be applicable within specific populations instead of across mixed populations. Finally, although the SNP HET rates could be inferred using linkage disequilibrium information (i.e., pair-wise linkage disequilibrium *r* ^2^), the estimation of *r* ^2^ and variance of *r* ^2^ themselves are subject to the effects of sample size and evolutionary history of specific SNPs [24]. Therefore, the sample size and variance of *r* ^2^ should be considered when *r* ^2^ are used for inferring SNP HET rates if a study has stringent requirements (i.e., development of clinical diagnostic markers).

We did not distinguish coding and non-coding regions of the genome in this simulation since Cargill et al. [5] reported that there is no significant difference in SNP density between coding and non-coding regions, and since the breakpoints of chromosome loss are poorly understood. We also examined the detection probability of LOH due to various sizes of chromosome loss assuming all known SNPs were used. This question is closely related to sample size and statistical power calculation in the experimental design for a neoplastic progression study; e.g., a small segment of chromosome loss has a lower detection probability for LOH, and in order to detect it, large sample sizes are needed. We also verified the simulation results directly using the SNP genotype data (all SNPs were used) from 90 individual subjects from the HapMap database (Figure 4, red line). The detection probabilities based on simulation methods are reasonably close to the observed data, but may be overly optimistic to a certain degree. Such differences may be due to bias in the SNP HET rate estimation distribution [25] toward common SNPs (Figure 1) or to a non-random distribution of SNPs.

Our study showed that a region of LOH greater than 200 kb could be detected with high probability (>90%), with losses smaller than 50 kb having a substantially lower detection probability when using all SNPs currently in the HapMap database (Figure 4). Higher densities of SNPs exist in certain chromosomal regions that provide the opportunity for reliably (*p* > 0.95 or 0.99) detecting LOH of segment sizes smaller than 50 kb (Figure 6). Finally, we evaluated the LOH detection probability for the given inter-SNP distances as reported for many commercial products (e.g., SNP-based genotyping arrays) or in published studies. For inter-SNP distances of 120 kb to 200 kb, the probability of detecting LOH for LOH of 300 kb or smaller ranges from 20% to 60% depending on SNP HET rates. The detection probability appears close to 1 if the region of loss is 900 kb or larger. The detection probabilities with inter-SNP distances 120 or 200 kb indicated in Figure 5 are substantially lower than the results shown in Figure 4 for a similar size of LOH. This is because the results in Figure 4 assume all SNPs currently reported in HapMap were used, whereas for Figure 5, SNPs with fixed inter-SNP distances (fewer SNPs) were used to calculate the detection probabilities. To increase detection probability, more SNPs should be used, or inter-SNP distance should be minimized (red line in Figure 5); however, this might be limited by the actual number of HET SNPs in a given chromosome segment. An alternate solution would be to increase sample size (statistical power) to detect small size of loss in an experiment. To a certain degree, improvements in LOH detection algorithms will increase the LOH detection probability. Improvements might include increasing the sensitivity of LOH detection in mixed cell populations (i.e., the neoplastic changes in somatic tissue). Combining copy number measurements and allele ratio measurements will increase detection of deletions but not copy neutral LOH. However, the resolution of LOH will still be constrained by the informative SNP distribution pattern itself. Sequencing or screening more human subjects to find more new SNPs could improve the theoretical detection probability as shown in Figures 4 and 6 only if the future-discovered SNPs are of great abundance and have high HET rates. For example, a SNP chip with 1 million SNPs to cover the 3 billion bp human genome would have a 3 kb mean inter-SNP distance. If the SNPs were evenly distributed throughout the genome to maximize coverage, the regions of LOH would need to be 32kb or larger in order to be detected with 0.95 probability assuming a SNP HET rate of 0.25 (and 26kb or larger for a HET rate of 0.3). Due to uneven distribution of SNPs in actual sequences, the detection probability will fluctuate with similar patterns shown in Figure 6.

Using dbSNP and HapMap data, this study evaluated the distribution of SNP HET rates and resolution of LOH genome wide. The results of this study have two important implications that might improve design and interpretation of future genome wide LOH screens of cancers and premalignant tissues. First, retrospective review of previous genome-wide LOH screens indicate that technology limitations (i.e., SNP density of arrays) used in the experiments could have missed significant numbers of LOH events that were below the resolution of the SNP array [26–31]. By using the analysis methods reported in this paper, reports of genome wide LOH could discuss the limitations of the resolution of the study in terms of what might have been missed in addition to the important loci that were discovered. A well-designed study using carefully selected SNP sets for evaluating specific regions on several chromosomes still had more than 280 kb distance on average between two informative SNPs [32]. However, in general, 280 kb is still relatively large considering an average gene size is 3 to 20 kb in the human genome, and smaller regions of LOH (i.e., <50 kb) might still be important, especially for early stages of neoplastic progression. The characteristics of LOH resolution mentioned above still apply to higher-density SNP arrays.

LOH has been frequently proposed as a candidate biomarker for cancer risk prediction. The ability to detect an LOH event will depend on informativity, SNP density, and the size of the LOH event. Our results could improve sample size calculations for design of future LOH studies. If one would like to detect the effect of an LOH event on the risk of progression to cancer, then the sample size depends on the LOH detection probability. For example, in a study with a 1:5 ratio of cases and controls, a minimum detectable relative risk of the LOH of 5, a statistical detection power 0.9, and an LOH prevalence rate of 30% among informative subjects, at least 23 cases and 117 controls will be needed if the LOH detection probability is 100% (large region loss or high density of informative SNPs). However, if the LOH detection probability is 0.7 or 0.3, for example, (e.g., a smaller loss event, or fewer informative SNPs), then at least 44 cases and 190 controls or 116 cases and 468 controls will be needed, respectively.

All the results obtained in this analysis are based on the assumption that heterozygous SNPs are required for detection of LOH. New technologies are emerging that could be used to detect chromosome copy number changes (including deletion) using homozygous SNPs with a reasonably high accuracy [33,34]. However, since LOH can result from mechanisms that do not change copy number [35,36], using copy number approaches can only yield a partial picture of the LOH status of a region of interest. Combining the analyses presented in this study and copy number could lead to a high level of reliability and a higher resolution in LOH detection for neoplastic progression research and biomarker development.

## Methods

### Data.

The data for SNPs HET rates were downloaded from dbSNP (build 126) (ftp://ftp.ncbi.nih.gov/snp/organisms/human_9606/database/organism_data/). HapMap SNP data for the human genome were downloaded from the HapMap Web site (July 2006 release) (http://www.hapmap.org/genotypes/). We only used the CEU population (Utah residents with Northern and Western European ancestry) data from HapMap. Our methods can easily be extended to other ethnic group data. The estimated SNP HET rates >0.5 were dropped from the analysis of HET rate distribution. The estimated variances for SNP HET rates were directly obtain from dbSNP.

### Simulation.

Data from dbSNP were used to summarize the HET rate distribution pattern of SNPs (Figure 1) and evaluate the estimated variances of HET rates in dbSNP (Figure 2). To estimate the number of SNPs needed for LOH detection in any given chromosomal region, a Monte Carlo simulation method was used. In this process, a SNP was selected and the determination of its heterozygosity was based upon the HET SNP distribution shown in Figure 1. This process was repeated until the cumulative probability of HET SNP reached the threshold at a predetermined *α* level (i.e., *α* = 0.05 or 0.01) which guarantees that the left-hand-side of Equation 2 will lie beyond the threshold (1 − *α*) 100% of the time (Figure 3). The simulation for chromosome segment deletion (Figure 4) was done using the genotype data from the HapMap CEU population data. In the simulation process, for each of the Chromosomes 1, 3, 9, 13, 17, and 18 (results of Chromosome 13 and 18 are unpublished data), a random segment was removed from the chromosome (mimicking the region of LOH on a chromosome), and the number of SNPs in the region was examined based on the genotype data of the individuals. The process was repeated 20,000 times for each segment size on a chromosome. The segment sizes of loss used in the simulation are: 5, 10, 20, 30, 50, 100, 200, 300, 500, 1,000, 2,000, 3,000, 4,000, and 5,000 kb. Based on these data, three methods (negative binomial model fitting, Monte Carlo simulation, and bootstrap) were used to investigate the relationship between the size of chromosome loss and probability of LOH detection. For negative binomial model fitting, which was found to fit the data best among the various theoretical distributions we evaluated, the discrete frequency distribution patterns of HET SNPs for each segment size listed above were fitted to a negative binomial model. Specifically, for the data of each segment size of loss, the HET SNP counts in each sample along a chromosome were used to estimate the parameters of negative binomial distribution with maximum likelihood method. The random numbers of HET SNPs were then generated based on the fitted negative binomial distribution parameters for each size of segment loss. This was repeated 10,000 times for each segment size and the detection probabilities were calculated based on the process for each segment (Figure 4, magenta lines). For the Monte Carlo simulation (Figure 4, blue and black lines), for each size of deletion listed above, the number of SNPs for each segment was counted, and the number of HET SNPs and detection probabilities were determined based on the empirical distribution pattern shown in Figure 1. For the bootstrap method, the observed detection probability (Figure 4 red line) was obtained by directly counting the HET SNPs in each segment based on the real genotyping data in the bootstrap sampling process. The results in Figure 5 were obtained by the probability model described in the text.

To examine the spatial pattern of LOH detection probability along a chromosome (Figure 6), we chose the 500 kb window size along Chromosomes 1, 3, 9, and 17, and within each window samples were randomly taken with various loss sizes to calculate the probabilities of LOH detection within each window along the chromosome. Similar patterns were found on other chromosomes (unpublished data). All analyses and simulations were carried out with Matlab (version 7.1, The MathWorks).

## Abbreviations

ASN: average sample number
HET: heterozygosity
LOH: loss of heterozygosity
SNP: single nucleotide polymorphism
SPRT: sequential probability ratio test
